# Uncovering the link between incidental physical activity and inhibition of automatic responses in aging. An ERP study

**DOI:** 10.3389/fnagi.2025.1602114

**Published:** 2025-08-01

**Authors:** Javier Sanchez-Lopez, Juan Silva-Pereyra, Sergio Manuel Sánchez-Moguel, Graciela Catalina Alatorre-Cruz, Mauricio González-López, Jorge A Sigg-Alonso, Mariana Pérez-Figueroa, Thalía Fernández

**Affiliations:** ^1^Escuela Nacional de Estudios Superiores Unidad Juriquilla, Universidad Nacional Autónoma de México, Querétaro, Mexico; ^2^Facultad de Estudios Superiores Iztacala, Universidad Nacional Autónoma de México, Estado de México, Mexico; ^3^Laboratorio de Psicofisiología, Departamento de Neurobiología Conductual y Cognitiva, Instituto de Neurobiología, Universidad Nacional Autónoma de México, Querétaro, Mexico; ^4^Unidad de Investigación en Neurodesarrollo, Departamento de Neurobiología Conductual y Cognitiva, Instituto de Neurobiología, Universidad Nacional Autónoma de México, Querétaro, Mexico; ^5^Escuela de Psicología, Universidad Anáhuac Querétaro, Querétaro, México

**Keywords:** cognitive reserve, incidental physical activity, inhibition of automatic responses, event-related potentials, aging

## Abstract

The concept of cognitive reserve explains how the brain maintains function despite age-related changes or neuropathological damage. Factors such as education, cognitive stimulation, and physical activity contribute to strengthening this reserve. While research has highlighted the benefits of structured exercise, less attention has been given to the impact of incidental physical activity (IPA) everyday, unplanned movements like walking or household chores. This study examined the relationship between IPA and the inhibition of automatic responses, a key executive function that tends to decline with age. A total of 59 healthy older adults (mean age = 67; standard deviation = 4.95; range = 60–82; 35 females) were assessed and divided into two groups based on their IPA levels, measured using the Yale Physical Activity Survey. They then completed a Counting-Stroop task, designed to assess inhibitory control, while event-related potentials (ERPs) were recorded to measure brain activity. Behavioral results confirmed the Stroop effect in both groups, with similar patterns observed overall and only one between-group difference during the incongruent condition. ERP analyses revealed greater late negativity as a result of the differences between conditions (1,050–1,200 ms) during the counting-Stroop task in the high-IPA group, suggesting more effective late-stage inhibitory processing post-execution likely related to re-evaluation and resolution of the conflict, while the low-IPA group lacked this effect. Furthermore, distinct neural activity patterns between the conditions were observed for each group as well. The high-IPA group showed differences between congruent and incongruent conditions between 300 and 500 ms, suggesting earlier conflict monitoring, while the low-IPA group exhibited significant differences over frontal areas in the 500–700 ms window, likely suggesting a different strategy for resolving interference. These findings suggest that IPA may enhance executive function by mainly supporting the later stages of inhibitory control mechanisms at a neural level, even when behavioral performance remains comparable. Given its accessibility, IPA may be a valuable strategy to maintain cognitive reserve and promote healthy aging. Future research is necessary to further explore the relationship between IPA and cognition in the context of cognitive reserve.

## 1 Introduction

Cognitive reserve refers to the capacity of the brain to maintain function and adapt to age-related changes or neuropathological damage by efficiently and flexibly utilizing cognitive resources or recruiting alternative neural networks as a compensatory mechanism (Barulli and Stern, 2013; Cabeza, 2002; Davis et al., 2008; Nithianantharajah and Hannan, 2009; Reuter-Lorenz and Park, 2014; Stern, 2002, 2009, 2017), to maintain high levels of cognitive functioning despite of these challenges (Chen and Nakagawa, 2023; López Sánchez and Granados, 2021; Meng and D’Arcy, 2012; Stern, 2012; Tucker and Stern, 2011). Previous studies have consistently shown that cognitive reserve is not a fixed trait but rather something that can be developed and enhanced over a lifetime (Barulli and Stern, 2013; Stern, 2002). Various lifestyle factors, such as education, occupation, cognitive stimulation, social interactions, and, notably, physical activity, have been identified as key contributors to building cognitive reserve (Stern, 2017; Valenzuela and Sachdev, 2006). Among these, physical activity has been particularly emphasized for its positive impact on brain health and cognition (for example, memory, attention, and executive functions; Bherer et al., 2013; Erickson et al., 2015; Sofi et al., 2011), been recognized as a key factor in promoting cognitive reserve (Chen and Nakagawa, 2023) through multiple mechanisms, such as the stimulation of brain-derived neurotrophic factor (Erickson et al., 2011) and the reduction of oxidative stress (Vinetti et al., 2015) and inflammatory response (Enokida et al., 2024; Frank et al., 2019; Nishida et al., 2015; Wärnberg et al., 2010), characteristic of aging (Franceschi and Campisi, 2014), as well as improvements in cerebral vascular function (Barnes et al., 2021). While age-related morphological changes in the brain, such as cortical thinning, white matter degradation, and hippocampal atrophy, are well-documented (Blinkouskaya et al., 2021), they do not consistently result in cognitive impairment. Although some benefits of physical activity may relate to structural brain maintenance (Raichlen and Alexander, 2017), increasing evidence also supports its role in enhancing functional compensatory mechanisms, which are key to cognitive reserve (e.g., Cabeza et al., 2018; Stern et al., 2020).

Most research on the relationship between physical activity and cognitive reserve has focused on structured exercise programs, that is, routines that involve deliberate, planned physical activities such as running, swimming, or resistance training. While these structured forms of exercise are undeniably beneficial (Colcombe and Kramer, 2003; Erickson et al., 2019; Sofi et al., 2011; Zhang et al., 2023), less attention has been given to the impact of incidental physical activity (IPA), which refers to unstructured, everyday movements that occur as a result of daily life choices (for example, walking to the store, gardening, taking the stairs, and household chores, among others; Shephard, 2013). IPA can contribute to overall health and wellbeing contributing to building cognitive reserve (Alatorre-Cruz et al., 2020; Andrews et al., 2022; Gongora-Meza and Sanchez-Lopez, 2025; Ross and McGuire, 2011; Sanchez-Lopez et al., 2018).

The focus of this article is to examine inhibitory control, specifically the inhibition of automatic responses, in older adults and its relationship with incidental physical activity. Inhibitory control refers to the ability to suppress irrelevant stimuli during goal-directed tasks (Bari and Robbins, 2013; Simpson and Carroll, 2019) and to differentiate relevant stimuli from distractors (Itti and Koch, 2001). The inhibition of automatic responses involves stopping an initiated action, ideally triggering a reactive inhibition mechanism (Aron, 2011). However, in some cases, participants engage in a proactive mechanism, anticipating the event before it occurs (Braver et al., 2009). These two mechanisms impose distinct demands on the executive control system (Littman and Takács, 2017) and share a common generative network, yet they are supported by different network operations. The reactive mechanism is strongly right-lateralized (Aron, 2011; Aron et al., 2004) and relies largely on activations in the fronto-parietal and ventral networks (Zhang et al., 2017). In contrast, the proactive mechanism more strongly engages the fronto-parietal network (Aron, 2011).

Therefore, the inhibition of automatic responses is a crucial component of executive control, (Diamond, 2013; Kipp-Harnishfeger, 1995; Logan, 1985) allowing to avoid impulsive actions and making reasoned decisions. inhibition of automatic responses is required when participants are asked to name the color of the ink in which a word is printed rather than reading the word itself, as in the classic Stroop task (Kipp-Harnishfeger, 1995; Portugal et al., 2018). Similarly, in the Counting-Stroop task, participants must inhibit the automatic tendency to read numerical digits and instead focus on counting the number of digits presented [e.g., saying “three” when presented with “2 2 2”; Sánchez-Moguel et al. (2018)]. Unlike the classic Stroop, where interference combines perceptual (e.g., color-word mismatch) and semantic conflicts, the Counting-Stroop isolates semantic interference, providing a more specific measure of inhibitory control (Bush et al., 1998; Xiao et al., 2011). Both tasks highlight the importance of inhibitory control in selective attention and the ability to override pre-potent responses. Some previous studies have proposed that inhibitory control tends to decline with age, contributing to difficulties in decision-making and impulse regulation among older adults (Carlson et al., 1995; Hasher et al., 1991), while other authors have reported no age-related deficit in inhibitory control and suggested that more studies are necessary to draw a firm conclusion about this association (Rey-Mermet and Gade, 2018; Rey-Mermet et al., 2018). A recent study reports that healthy older adults displaying electrophysiological markers associated with risk for cognitive decline (i.e., an excess of theta activity during resting EEG, as compared to a normative database) show a lack of conflict detection [i.e., the detection and resolution of competing stimulus or response representations, according to Larson et al. (2014)] during inhibition tasks compared to those without such risk (Sánchez-Moguel et al., 2018).

One of the ways to measure cognitive processing during the Stroop task is through event-related potentials, as they allow us to track the precise timing of neural processes involved in cognitive control and attention. Their value lies in their ability to reveal underlying neural mechanisms that may not be evident just through behavioral measures, providing a more comprehensive understanding of cognitive processing during interference tasks. Among the main components of the ERPs typically observed during the performance of the Stroop task (Sánchez-Moguel et al., 2018; West and Alain, 1999, 2000), the N450 in young adults or N500 in older adults; is a negative deflection occurring around 450/500 ms post-stimulus, which has been associated with conflict detection and cognitive control processes, reflecting the response of the brain to incongruent stimuli. The P300 component, a positive deflection peaking approximately 300 ms after the stimulus onset, is linked to attention and stimulus evaluation mechanisms, often exhibiting increased amplitude in response to infrequent or significant stimuli. These ERPs provide pivotal insights into the temporal dynamics of cognitive processing during tasks involving interference and attentional control (Sánchez-Moguel et al., 2018; West and Alain, 1999). Additionally, earlier components such as the N200, observed between 150 and 300 ms (Sánchez-Moguel et al., 2018), have been implicated in the initial stages of conflict monitoring, while later activity between 1,060 and 1,160 ms post-stimulus (West and Alain, 1999) has also been described, potentially reflecting extended cognitive processing in older adults. Although these are not considered classical Stroop-related components, they have been examined in the context of aging and may provide further insight into the temporal dynamics of cognitive control across the lifespan. However, their specific role within Stroop task performance has not yet been well-characterized, highlighting the need for further research to elucidate their functional significance in this context.

Previous studies have proposed that physical activity enhances inhibitory control in physically active older adults compared to those who are inactive (Feng et al., 2024). For instance, a previous study demonstrated that physically active individuals, compared to sedentary ones, exhibited faster reaction times, greater accuracy, and reduced variability in the Stroop task, along with lower P2 latency and reduced frontocentral N2 and N450 amplitudes, suggesting enhanced attentional control and reduced interference processing (Gajewski and Falkenstein, 2015). Other study in this field (Jintian, 2019) have demonstrated that older adults engaging in high-intensity physical activity show faster response times, greater attentional resource allocation, and improved information processing speed during interference control tasks like the Stroop test, compared to sedentary peers. These active older adults appear to develop enhanced frontal lobe compensatory mechanisms that help to maintain cognitive processing efficiency at levels comparable to younger individuals (Jintian, 2019). However, additional research has failed to replicate these performance benefits, showing no significant effects of physical activity on Stroop task outcomes. This growing body of work presents inconclusive evidence regarding the precise relationship between physical activity, Stroop performance, and its neural correlates (Gajewski et al., 2020).

Furthermore, research has highlighted that open-skilled physical activities, such as dancing, are associated with improvements in inhibition, visual tracking, and cognitive flexibility in older adults (Ingold et al., 2020). Additionally, a study suggested that even a single bout of moderate exercise can enhance inhibitory control and working memory functions in healthy older adults (Martini et al., 2024). These findings suggest that the practice of physical activity may play a critical role in maintaining and improving cognitive functions, particularly inhibitory control, in aging populations. Even though a few studies have suggested the relationship between IPA and brain electrical activity during rest (Sanchez-Lopez et al., 2018) and task (Alatorre-Cruz et al., 2020), the connection between IPA and inhibition of automatic responses remains underexplored.

Therefore, the present study aimed to investigate the association between the level of incidental physical activity and the ability to inhibit automatic responses in older adults as well as to explore the neurobiological correlates of this relationship using event-related potentials (ERPs). We hypothesize that among cognitively healthy older adults, those with higher levels of incidental physical activity will exhibit superior behavioral performance in tasks requiring the inhibition of automatic responses. This enhanced performance is expected to be accompanied by ERP signatures indicative of more efficient cognitive control. Specifically, we anticipate greater amplitude differences between congruent and incongruent conditions in components related to conflict monitoring and response inhibition (e.g., N200, P3, N500, and/or late processing components), reflecting more robust discrimination of task-relevant stimuli and more effective allocation of attentional resources, compared to their peers with lower levels of incidental physical activity. Understanding how lifestyle factors, such as IPA, associates with the ability to inhibit automatic responses could provide valuable insights into strategies for promoting healthy cognitive aging and enhancing cognitive reserve. Incidental movement might offer a low-effort, higher motivation, and an accessible way to bolster cognitive control functions in older adults, potentially delaying cognitive decline.

## 2 Materials and methods

### 2.1 Participants

Sixty-six older adults were invited to participate in the study using a non-probabilistic convenience sampling method. Following an initial screening process based on predefined inclusion and exclusion criteria (detailed below), the final sample included 59 participants (35 females) older than 60 years (mean age = 67.16 years, SD = 4.95).

The inclusion criteria required participants to have at least 9 years of schooling and a score above 90 on the Spanish version of the Wechsler Adult Intelligence Scale-WAIS (Wechsler, 2012). None of the participants faced significant socioeconomic disadvantages, as assessed by the Regla AMAI NSE 8 × 7 (AMAI, 2011). Participants were also evaluated to ensure that they had an adequate quality of life based on the Quality-of-Life Enjoyment and Satisfaction Questionnaire (Q-LES-Q; Endicott et al., 1993). Lifestyle factors were evaluated through a survey covering habits such as attending workshops, learning and using new languages, adopting new technologies, consuming in news, engaging hobbies (e.g., reading, writing, listening to or playing music), traveling, attending cultural events, and participating in social, cultural, or religious activities. All participants scored within normal ranges on the Spanish neuropsychological test battery NEUROPSI (Ostrosky-Solis et al., 1999), which evaluates cognitive functions like orientation, attention, memory, language, visuoperceptual skills, and executive functions.

Participants underwent a clinical evaluation by a geriatric psychiatrist to rule out psychiatric or neurological disorders as well as ensure that no symptoms of cognitive decline were evident. Clinical blood analyses confirmed that none of the participants had uncontrolled medical conditions such as anemia, diabetes, hypercholesterolemia, or thyroid disease; none of them had uncontrolled hypertension.

Participants completed a counting-Stroop task while their event-related potentials (ERPs) were recorded (see below). We ensured that all participants performed above the chance level (> 56.6% correct responses); no individuals were excluded from the final analysis based on this criterion. The average accuracy for the sample was 81.5% (SD = 10.4). Participants were divided into two groups: high-level incidental physical activity (h-IPA) and low-level incidental physical activity (l-IPA), this classification is described in the next section. Seven participants (three from the l-IPA group and four from the h-IPA group) reported engaging in recreational sports, including tennis, running, chess, volleyball, and swimming. A two-way chi-squared test revealed no significant differences in recreational sport participation between groups (Pearson χ^2^ = 0.41, *p* = 0.52).

The study was approved by the Bioethics Committee of the Instituto de Neurobiología at the Universidad Nacional Autónoma de México. Participants provided informed consent and were incentivized with free access to their clinical screening results and recommendations to enhance their lifestyle-related health. The research was conducted at the Laboratorio de Psicofisiología of the Instituto de Neurobiología at the Universidad Nacional Autónoma de México.

### 2.2 Group classification by incidental physical activity level

As in previous studies (Alatorre-Cruz et al., 2020; Sanchez-Lopez et al., 2018), this database was divided into two groups: high-level incidental physical activity (h-IPA) and low-level incidental physical activity (l-IPA). To do this, a k-means cluster analysis was performed to group participants based on the total physical activity index of the Yale Physical Activity Survey (YPAS; see below). The analysis was performed with the number of clusters set to two, as the research question required a clear distinction between high and low activity levels. The initial cluster centers showed large differences: cluster 1 had a high total physical activity index (Mean = 105), while cluster 2 had a significantly lower value in that same variable (Mean = 11). The iteration history indicated that the algorithm converged after two iterations, meaning the cluster centers stabilized and no further changes were needed. The final cluster centers also demonstrated distinct groupings: cluster 1 was also characterized by a high total physical activity index (Mean = 73), while cluster 2 represented participants with a lower index (Mean = 38). The distance between the centers of the final cluster was 34.75, suggesting a separation between the two groups. The ANOVA results indicated significant differences between the groups [F(1, 57) = 104.74, *p* < 0.001]. The final solution assigned 27 cases to Cluster 1 (h-IPA) and 32 cases to Cluster 2 (l-IPA), with no missing data.

**FIGURE 1:**
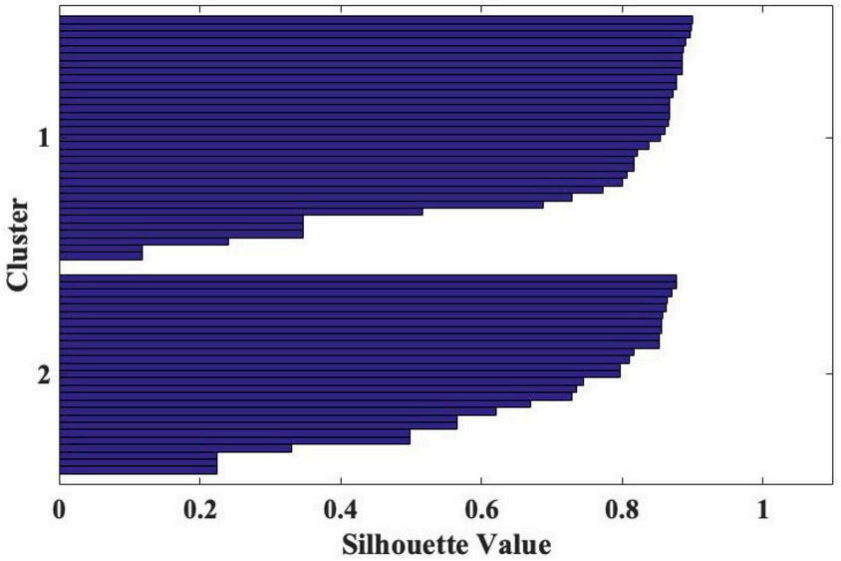
Two clusters as result of cluster silhouette analysis, using Euclidean distance as a measure of dissimilarity.

To assess the clustering quality, we conducted a cluster silhouette analysis using Euclidean distance as a measure of dissimilarity. The results revealed that Cluster 1 had a mean silhouette score of 0.552 (min = 0.241, max 0.691), while Cluster 2 had a mean score of 0.562 (min = 0.069, max = 0.715). Across the entire sample (59 cases), the total mean silhouette score was 0.558, indicating a moderate level of cluster cohesion and separation. These results demonstrate that the identified groups are balanced in size and show acceptable quality, as indicated by their silhouette values shown in Figure 1.

As mentioned above, this statistical analysis considered variables from the YPAS (De Abajo et al., 2001), including total kcal/week, the vigorous activity index, and the moving index. The YPAS assesses physical activity levels in adults aged 60 and older through a self-report questionnaire consisting of two parts. The first part gathers information on the time spent on activities such as housework, employment, yard work, caregiving, and leisure pursuits. The second part focuses on the frequency and duration of vigorous activities, leisurely walking, standing, moving, and sitting. After participants complete the questionnaire, the evaluator processes the data using score sheets provided in the YPAS. For the first section, metrics such as time in minutes per week, energy expenditure in metabolic equivalents (MET) by time, and energy expenditure in kilocalories (calculated as MET × time × weight) are determined for each activity, along with domain-specific and total values. In the second section, partial indices are derived for each activity by multiplying the frequency score by its duration and then by a weighting factor that reflects the relative intensity of the activity, as specified in the questionnaire. The total index is obtained by summing these partial indices.

Table 1 presents demographic, neuropsychological blood analysis, and YPAS scores data of the h-IPA and l-IPA. Notably, significant differences between groups were only found in diverse domains of the YPAS energy expenditure and YPAS index scores.

**TABLE 1:** Characteristics of the participants by group for demographic variables, WAIS, NEUROPSI, quality of life, lifestyle, blood analysis, and YPAS.

Variable	h-IPA group	l-IPA group	Statistical comparison		
	Female/male	Female/male	Pearson χ^2^	P	
Gender	17/10	18/14	0.27	0.60	
	Mean (SD)	Mean (SD)	t(57)	*P*	Cohen’s d
Age	66.14 (3.91)	68.03 (5.59)	1.47	0.14	−0.39
Years of schooling	15.11 (4.10)	15.82 (4.68)	0.61	0.53	−0.16
BMI	24.30 (2.84)	25.77 (2.87)	1.96	0.054	−0.51
Socioeconomic level	215.70 (33.67)	217.84 (29.08)	0.26	0.79	−0.07
NEUROPSI	108.57 (7.80)	106.92 (8.07)	0.79	0.43	0.21
Quality of life	78.17 (8.67)	78.44 (10.11)	0.10	0.91	−0.03
Lifestyle survey	128.14 (68.92)	143.81 (74.55)	0.83	0.40	−0.22
*WAIS IQs*					
Total IQ	104.22 (7.67)	103.15 (9.36)	0.47	0.63	0.12
Verbal IQ	110.48 (7.79)	110.37 (7.01)	0.05	0.95	0.01
Performance IQ	100.81 (8.16)	99.00 (12.41)	0.65	0.51	0.17
WAIS indices					
VCI	122.85 (9.34)	122.12 (9.34)	0.30	0.75	0.08
POI	105.14 (12.40)	105.68 (15.42)	0.14	0.88	−0.04
WMI	103.59 (5.02)	103.31 (4.39)	0.22	0.82	0.06
PSI	108.37 (15.59)	102.31 (20.92)	1.24	0.22	0.32
*Blood analysis*					
Total cholesterol	185.59 (38.25)	194.68 (35.36)	0.94	0.34	−0.25
Hemoglobin	14.66 (1.02)	15.30 (1.23)	1.99	0.051	−0.56
Glucose	96.74 (11.10)	99.37 (19.53)	0.62	0.53	−0.16
Thyroid-stimulating hormone	2.33 (1.15)	2.21 (1.45)	0.36	0.71	0.09
Triglycerides	123.01 (61.95)	126.62 (61.09)	0.22^#^	0.82	−0.06
Anterogenic index	3.79 (1.42)	4.21 (1.11)	1.25^#^	0.21	−0.33
*YPAS kcal/week*					
Housework	5772.02 (3456.18)	3410.91 (1825.22)	**3.35**	**0.001**	**0.88**
Work	542.43 (1351.51)	671.19 (2559.99)	0.23	0.81	−0.06
Yardwork	479.24 (535.67)	352.49 (401.79)	1.03	0.30	0.27
Caretaking	1501.44 (2775.28)	1400.56 (3103.11)	0.13	0.89	0.03
Leisure	4109.10 (3136.43)	2377.95 (1671.00)	**2.70**	**0.009**	**0.71**
*YPAS index*					
Vigorous activity	40.85 (15.66)	13.84 (13.47)	**7.12**	**< 0.001**	**1.86**
Leisure walking	12.88 (9.03)	10.21 (9.07)	1.12	0.26	0.29
Moving	11.77 (3.10)	7.87 (4.15)	**4.02**	**< 0.001**	**1.05**
Standing	4.22 (3.56)	3.18 (2.37)	1.33	0.18	0.35
Sitting	2.59 (0.79)	3.12 (1.12)	**2.05**	**0.04**	−**0.54**

^#^t(56) blood analysis values for one participant of l-IPA group are missing. Statistical comparisons between groups are also shown. BMI, body mass index; NEUROPSI, brief neuropsychological test battery in Spanish; WAIS, Weschler Adult Intelligence Scale; IQ, intelligence quotient; VCI, verbal comprehension index; POI, perceptual organization index; WMI, working memory index; PSI, processing speed index; YPAS, Yale Physical Activity Survey. Significant differences are indicated in bold.

### 2.3 Counting-Stroop task

Based on a previous study (Sánchez-Moguel et al., 2018), a list of one, two, three, or four words denoting one of the following numbers in Spanish (“uno/one,” “dos/two,” “tres/three,” “cuatro/four”) was displayed at the center of a 17-inch computer screen. Each stimulus was shown for 500 ms, with an inter-stimulus interval of 1.5 s. In the incongruent condition, the number of words presented did not match the numerical meaning of the word itself (e.g., “dos dos dos dos/two two two two,” where four words are presented, but the numbers refer to “two”), while in the congruent condition, the number of words matched the meaning of the word (e.g., “tres tres tres/three three three,” where three words are presented, all referring to the number “three”). A total of 120 incongruent and 120 congruent stimuli were presented in random order.

Participants were comfortably seated in front of the screen and were instructed to indicate how many times the word appeared in each trial using a response pad held in their hands. Half of the participants used their left thumbs to respond to “one” or “two” and their right thumbs for “three” or “four,” while the other half used the opposite hands to counterbalance motor responses. Participants were encouraged to respond as quickly and accurately as possible. To ensure an understanding of the task, a brief practice session was conducted before the main experiment. Event-related potentials were recorded during the task performance.

### 2.4 Event-related potentials acquisition and preprocessing

The EEG was recorded using 32 Ag/AgCl electrodes embedded in an elastic cap (Electrocap), with NeuroScan SynAmps amplifiers (Compumedics NeuroScan) and Scan 4.5 software (Compumedics NeuroScan). The electrodes were referenced online to the right earlobe (A2), while the left earlobe (A1) was recorded as another active channel. The recordings were later re-referenced offline to the average of both earlobes. The EEG data were digitized at a sampling rate of 500 Hz, with a band-pass filter set between 0.01 and 100 Hz. Electrode impedances were maintained below 5 kΩ. Additionally, an electro-oculogram was recorded using a supraorbital electrode and another electrode placed on the outer corner of the left eye.

Event-related potentials (ERPs) were obtained for each participant and experimental condition (congruent and incongruent). Epochs of 1,500 ms were extracted for each trial, comprising a 200 ms pre-stimulus interval and a 1,300 ms post-stimulus interval. An eye-movement correction algorithm was applied to eliminate artifacts from blinks and vertical eye movements (Gratton et al., 1983). Offline processing included low-pass filtering at 50 Hz with a 6-dB slope. Baseline correction was performed using the 200 ms pre-stimulus window, and a linear detrend correction was applied to the entire epoch. Epochs with voltage fluctuations exceeding ± 80 μV were automatically excluded from the final average. The epochs were also visually inspected, and any epochs containing visible artifacts were discarded. No electrodes were removed or interpolated. The averaged waveforms for each participant and condition were based solely on trials with correct responses. The mean of segments included in the averaged waveforms was the same between conditions and did not differ between groups (mean l-IPA = 92.15; mean h-IPA = 92.22; t(57) = 0.016; *p* = 0.98).

### 2.5 Statistical analysis

#### 2.5.1 Counting-Stroop task behavioral analysis

Four separate mixed-design 2-way ANOVAs were conducted to analyze the percentage and transformed percentage of correct responses, mean reaction time of correct responses and reaction time variability (calculated as the standard deviation of the reaction times for correct responses). The between-subject factor was group (h-IPA and l-IPA), while the within-subject factor was condition (congruent and incongruent). A transformed percentage of correct responses was performed to ensure a normal distribution of the data by using the function {ARCSINE [Square Root (percentage/100)]} (McDonald, 2009). Additionally, Tukey’s honest significant difference (HSD) post-hoc tests were performed for multiple comparisons. To further investigate group differences by condition, each condition (congruent and incongruent) was compared between groups across the measures: accuracy, transformed accuracy, reaction times, and reaction time variability. These comparisons are presented solely for descriptive purposes and are not intended to support inferential conclusions beyond the observed tendencies within each group.

#### 2.5.2 Counting-Stroop task event-related potentials analysis

Based on previous studies (Sánchez-Moguel et al., 2018; West and Alain, 1999) and by visual inspection of the ERPs for each condition across groups, as well as the difference waves (incongruent minus congruent condition) per group, four-time ranges corresponding to four ERP components were selected for further statistical analysis: 150–300, 300–500, 500–700, and 1,050–1,200 ms. The selection of time windows based on prior literature was intended to align with the criterion of *a priori* measurement parameter selection, as recommended by Luck and Gaspelin (2017). At the same time, visual inspection was used to account for potential variations in component latency which, as noted by Luck and Gaspelin (2017), may render time windows from previous studies inappropriate for a new experimental context. The statistical analysis aimed to investigate, first, the differences in the Stroop effect between groups through comparisons of the difference wave, calculated for each participant, between the h-IPA and l-IPA groups; and second, the presence of an ERP-related Stroop effect in each group, categorized by IPA level, separately which allowed for insights into each group’s functioning and their differential neural processing patterns. These analyses were done by comparing the mean amplitude values of the specified ERP components of the difference waves between groups for the first analysis, and the same ERP intervals between congruent and incongruent conditions in each group separately for the second analysis, across all scalp electrodes, using a non-parametric permutation *t*-test (2,000 permutations) with FDR correction for multiple comparisons (31 per analysis) using all the single trials from each subject. This method shuffles group or condition labels across trials to generate a null distribution, from which the exact probability (*p*-value) of observing the experimental effect by chance is estimated. This approach provides robust inference for ERP data. This data-driven method reduces the risk of selection bias and increases the sensitivity to detect effects that may not strictly conform to canonical topographies, especially when working with aging populations where component distributions may vary (Groppe et al., 2011; Pernet et al., 2015). All statistical analyses were conducted using the EEGLab STUDY toolbox (Delorme and Makeig, 2004).

## 3 Results

### 3.1 Behavioral results

Behavioral results are shown in Table 2, where all scores exhibited significant differences between conditions (incongruent and congruent). These results confirm the presence of a Stroop effect, as evidenced by higher accuracy, faster reaction times, and lower reaction time variability in the congruent condition compared to the incongruent condition, regardless of the group. No significant differences were found for the main effect of the group or the interaction. A marginally significant difference in reaction times was observed between groups (*p* = 0.056), regardless of the condition, suggesting faster reaction times in the l-IPA group. An additional analysis was conducted to explore potential differences between groups for each condition separately. The results revealed a significant difference in reaction time between groups only in the incongruent condition (p = 0.037), with l-IPA individuals responding faster than h-IPA individuals.

**TABLE 2:** Counting-Stroop task behavioral results.

Variable	h-IPA (*n* = 27)		l-IPA (*n* = 32)		F value (df = 1, 57)		*P*-value	Eta square
	*Cong (SD)*	*Incong (SD)*	*Cong (SD)*	*Incong (SD)*				
Accuracy (%)	85.03 (9.05)	76.72 (12.37)	87.34 (8.28)	77.00 (14.12)	Group	0.22	0.639	0.004
					**Condition**	**76.67**	**< 0.001**	**0.574**
					Interaction	0.91	0.342	0.016
Accuracy (ARCSINE)	1.19 (0.13)	1.08 (0.15)	1.23 (0.13)	1.09 (0.18)	Group	0.356	0.553	0.006
					**Condition**	**97.63**	**< 0.001**	**0.631**
					Interaction	1.08	0.301	0.019
Reaction times (mean)	662.98 (57.25)	729.76 (58.43)	637.69 (58.62)	695.45 (63.95)	Group	3.81	0.056	0.063
					**Condition**	**347.81**	**< 0.001**	**0.859**
					Interaction	1.82	0.182	0.031
Reaction times (variability)	102.54 (11.96)	106.92 (9.80)	101.17 (17.89)	108.62 (14.47)	Group	0.002	0.960	0.000
					**Condition**	**13.68**	**< 0.001**	**0.194**
					Interaction	0.920	0.341	0.016
**Between groups comparisons per condition**								
**Variable**	**h-IPA (*n* = 27)**		**l-IPA (*n* = 32)**		**t(df)**		***P*-value**	**Cohen’s d**
*Accuracy (%)*								
Congruent	85.03 (9.05)		87.34 (8.28)		1.02 (57)		0.310	−0.27
Incongruent	76.72 (12.37)		77.00 (14.12)		< 1 (57)		0.938	−0.02
***Accuracy (ARCSINE)***								
Congruent	1.19 (0.13)		1.22 (0.12)		1.02 (57)		0.308	−0.24
Incongruent	1.08 (0.15)		1.09 (0.17)		< 1 (57)		0.832	−0.06
*Reaction times (mean)*								
Congruent	662.97 (57.25)		637.68 (58.62)		1.66 (57)		0.101	−0.44
Incongruent	729.76 (58.43)		695.44 (63.95)		**2.13 (57)**		**0.037**	−**0.56**
*Reaction times (variability)*								
Congruent	102.53 (11.96)		101.17 (17.89)		< 1 (57)		0.737	−0.09
Incongruent	106.91 (9.80)		108.61 (14.47)		< 1 (54.62)		0.595	−0.14

h-IPA, high levels of incidental physical activity; l-IPA, low levels of incidental physical activity Cong, congruent condition; Incong, incongruent condition; DE, standard deviation; df, degrees of freedom. Significant differences are indicated in bold.

### 3.2 Event-related potentials results

**FIGURE 2:**
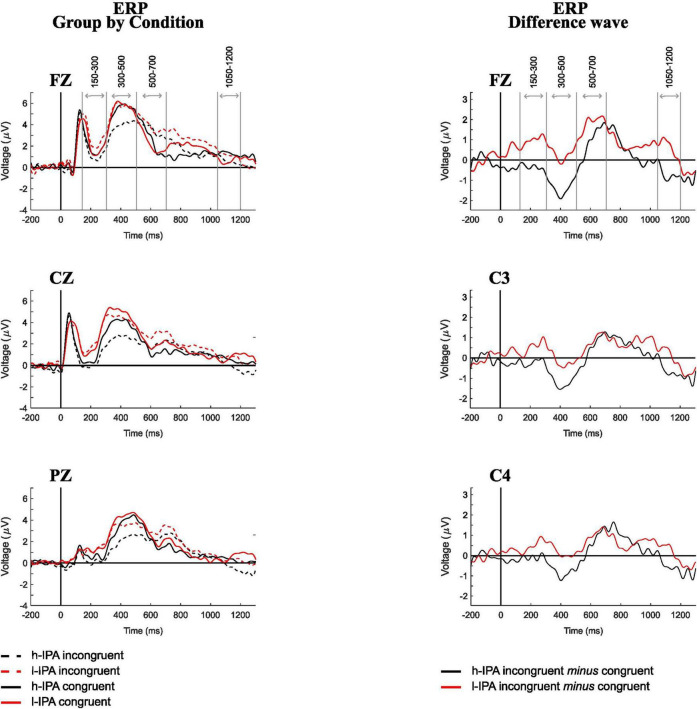
Event-related potential recorded during the counting-Stroop task for both conditions and groups. Left panel displays ERP by condition for FZ, CZ, and PZ in h-IPA and l-IPA groups. Right panel shows the ERP difference wave (incongruent minus congruent) for FZ, C3, and C4.

Event-related potential waves for each group and condition (Fz, Cz, and Pz), as well as the difference waves (incongruent *minus* congruent; Fz, C3, and C4), are shown in the left and right panels of Figure 2, respectively. These electrodes are displayed to illustrate the presence of the identified components. As is standard practice in ERP research, we conducted an initial visual inspection of the waveforms to guide the interpretation and definition of time windows. During the visual inspection of the ERPs for group and condition separately, a negative peak was observed between 150 and 300 ms, a positive peak between 300 and 500 ms, a negative peak between 500 and 700 ms, and a slow negative wave between 1,050 and 1,200 ms (indicated by gray lines in Figure 2). These observations served to contextualize subsequent statistical analyses. Differences in component morphology across conditions and groups were noted descriptively: for example, the l-IPA group showed larger positive deflections between 150–300, 500–700 and 1,050–1,200, while the h-IPA group exhibited larger negative peaks between 300–500 and 1,050–1,200 ms.

#### 3.2.1 Comparisons of the difference wave (incongruent *minus* congruent) between groups

**FIGURE 3:**
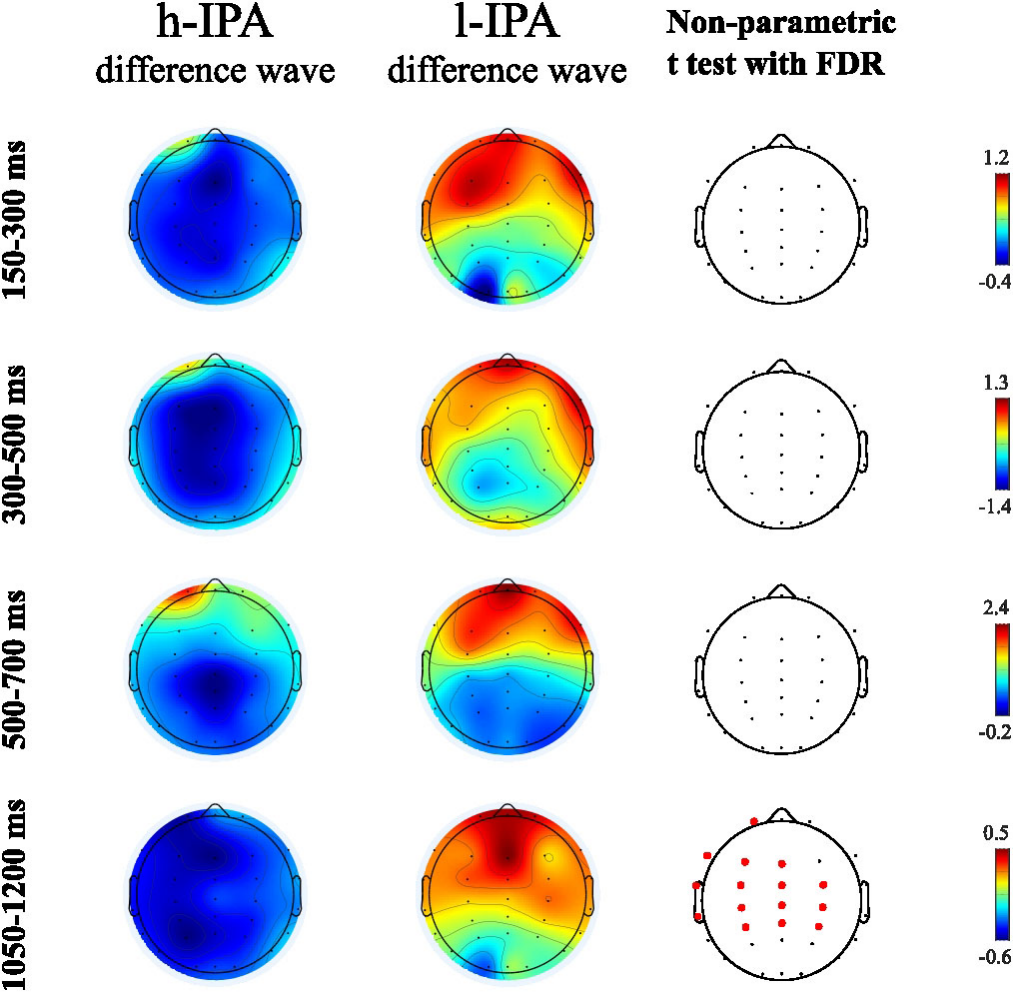
Topographic maps showing brain activity for h-IPA and l-IPA difference waves (incongruent minus congruent) at different time intervals (150–300 ms, 300–500 ms, 500–700 ms, 1050–1200 ms) in the first two columns. The third column displays results from a non-parametric t-test with FDR, indicating significant areas with red dots at the 1050–1200 ms interval. Color scales are shown for each map.

This first analysis aimed to evaluate differences between groups in the ERP effects related to the counting-Stroop task. Difference waves were calculated for all participants and the resulting ERP were statistically compared between groups. Among the ERP intervals analyzed, significant differences were found only in the mean amplitude between 1,050 and 1,200 ms, with a higher negative amplitude in the difference wave of the h-IPA group compared to the l-IPA group which, by contrast, exhibited a positive deflection. This difference was observed over parietal, central, and frontal electrodes, primarily in the left hemisphere (see Figure 3 and Table 3).

**TABLE 3:** Event-related potential statistical results for the time windows where significant differences were found.

Electrode	Between groups			Within group								
				h-IPA						l-IPA		
	1050–1200 ms			300–500 ms			1,050–1,200 ms			500–700 ms		
	t(df)	*P*-value	FDR *p*-value (31)	t(df)	*P*-value	FDR *p*-value (31)	t(df)	*P*-value	FDR *p*-value (31)	t(df)	*P*-value	FDR *p*-value (31)
FP1	2.24 (5400)	0.025	**0.046**	0.19 (5215)	0.717	1.000	1.80 (4587)	0.038	0.053	2.42 (6251)	< 0.001	**0.003**
FP2	1.80 (4320)	0.081	0.084	0.93 (4812)	0.567	1.000	1.76 (4779)	0.093	0.089	2.20 (6256)	0.003	**0.011**
F3	2.72 (4465)	0.008	**0.034**	4.31 (5162)	< 0.001	**< 0.001**	3.77 (5250)	< 0.001	**< 0.001**	3.19 (6275)	< 0.001	**< 0.001**
F4	1.87 (4321)	0.078	0.084	3.11 (5232)	0.017	**0.027**	3.31 (5249)	0.001	**0.002**	2.82 (6226)	< 0.001	**< 0.001**
C3	2.81 (5039)	0.010	**0.023**	4.71 (5170)	< 0.001	**< 0.001**	3.66 (5259)	< 0.001	**< 0.001**	1.05 (6293)	0.116	1.000
C4	2.76 (5080)	0.012	**0.023**	3.28 (5232)	0.006	**0.027**	2.88 (5258)	0.003	**0.007**	1.44 (6271)	0.062	0.095
P3	2.10 (5157)	0.036	0.051	4.56 (5169)	< 0.001	**< 0.001**	4.24 (5251)	< 0.001	**<0.001**	0.45 (6300)	0.381	1.000
P4	1.91 (5151)	0.059	0.073	3.73 (5183)	0.001	**0.010**	3.41 (5260)	< 0.001	**0.002**	0.59 (6287)	0.314	1.000
O1	0.20 (3555)	0.879	1.000	3.01 (5238)	0.016	**0.031**	3.60 (5232)	< 0.001	**0.002**	0.73 (6301)	0.295	1.000
O2	1.42 (5012)	0.175	1.000	2.79 (5228)	0.029	**0.042**	3.33 (5240)	< 0.001	**0.003**	0.53 (6239)	0.428	1.000
F7	2.60 (4428)	0.007	**0.043**	2.16 (5200)	0.080	0.095	3.59 (5259)	0.001	**< 0.001**	1.88 (6298)	0.005	**0.018**
F8	2.02 (4275)	0.058	0.064	2.24 (5144)	0.089	1.000	2.50 (5249)	0.008	**0.016**	3.70 (6184)	< 0.001	**<0.001**
T3	2.81 (4699)	0.005	**0.023**	2.05 (5184)	0.112	1.000	3.64 (5220)	< 0.001	**<0.001**	2.37 (6286)	0.014	**0.033**
T4	1.84 (4013)	0.072	0.084	1.81 (5247)	0.340	1.000	2.90 (5247)	0.001	**0.007**	1.84 (6261)	0.028	**0.042**
T5	1.84 (4895)	0.061	0.084	2.98 (5211)	0.008	**0.027**	3.64 (5223)	< 0.001	**<0.001**	1.33 (6282)	0.117	1.000
T6	1.07 (4776)	0.299	1.000	1.97 (5227)	0.204	1.000	2.48 (5227)	0.006	**0.016**	0.46 (6271)	0.450	1.000
CZ	2.28 (5169)	0.029	**0.046**	4.70 (5162)	< 0.001	**< 0.001**	2.74 (5256)	0.004	**0.007**	0.96 (6237)	0.138	1.000
FZ	4.07 (4907)	< 0.001	**< 0.001**	4.20 (5192)	< 0.001	**< 0.001**	3.93 (5224)	< 0.001	**< 0.001**	2.56 (6299)	< 0.001	**<0.001**
PZ	1.77 (5267)	0.085	0.084	4.76 (5209)	< 0.001	**< 0.001**	3.61 (5259)	< 0.001	**0.003**	0.78 (6284)	0.211	1.000
FCZ	3.23 (5023)	0.004	** < 0.001**	4.83 (5167)	< 0.001	**< 0.001**	3.28 (5250)	0.001	**< 0.001**	2.09 (6279)	0.002	**0.003**
CPZ	2.25 (5241)	0.026	**0.046**	4.59 (5199)	0.001	** < 0.001**	3.30 (5258)	< 0.001	**0.004**	0.87 (6247)	0.198	1.000
CP3	2.54 (5109)	0.018	**0.031**	4.70 (5165)	< 0.001	**< 0.001**	3.96 (5258)	< 0.001	**< 0.001**	0.68 (6293)	0.260	1.000
CP4	2.58 (5117)	0.017	**0.023**	3.53 (5214)	0.003	**0.024**	3.27 (5260)	0.001	**0.003**	0.97 (6277)	0.155	1.000
FC3	3.25 (4849)	0.002	**0.010**	4.66 (5153)	< 0.001	**< 0.001**	3.78 (5258)	< 0.001	**< 0.001**	2.76 (6291)	< 0.001	**< 0.001**
FC4	2.44 (4860)	0.025	**0.044**	3.58 (5230)	0.002	**0.010**	3.06 (5255)	0.002	**0.003**	2.61 (6251)	< 0.001	**0.003**
TP7	2.16 (4759)	0.035	0.057	2.50 (5208)	0.038	0.087	3.71 (5221)	< 0.001	**< 0.001**	1.52 (6301)	0.088	1.000
TP8	1.56 (4496)	0.125	1.000	1.63 (5254)	0.411	1.000	2.41 (5209)	0.014	**0.020**	0.85 (6267)	0.237	1.000
FPZ	2.26 (4435)	0.026	0.051	1.05 (5122)	0.390	1.000	1.99 (5188)	0.038	0.052	3.03 (6283)	< 0.001	**< 0.001**
OZ	1.76 (5169)	0.083	0.084	2.77 (5233)	0.035	**0.048**	3.38 (5239)	< 0.001	**0.003**	0.92 (6264)	0.171	1.000
FT7	2.90 (4748)	0.006	**0.023**	2.02 (5223)	0.094	1.000	3.51 (5247)	< 0.001	**< 0.001**	2.64 (6242)	0.011	**0.022**
FT8	1.87 (4130)	0.074	0.084	1.84 (5255)	0.281	1.000	2.54 (5257)	0.012	**0.014**	2.82 (6227)	< 0.001	**0.003**

Both between and within group analysis are shown. t, t-value; df, degrees of freedom; *P*-value, uncorrected *p*-value; FDR *p*-value (31), FDR corrected *p*-value over 31 comparisons. Significant differences are indicated in bold.

#### 3.2.2 Comparisons between incongruent and congruent conditions by group

**FIGURE 4:**
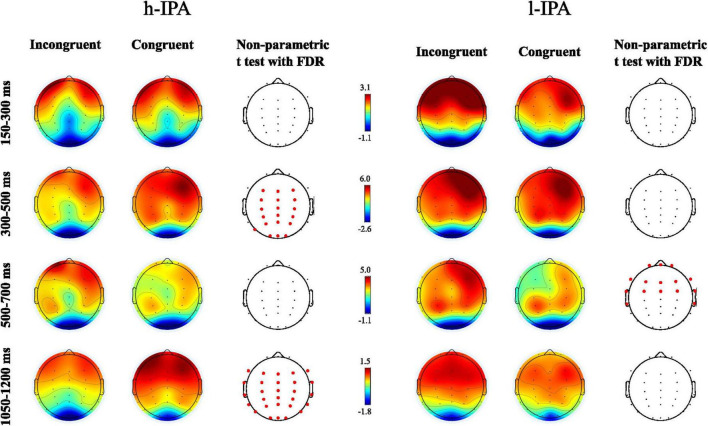
Results of the statistical non-parametric comparisons between congruent and incongruent conditions for each group separately (h-IPA on the left and l-IPA on the right). Four time intervals are presented: 150–300 ms, 300–500 ms, 500–700 ms, and 1050–1200 ms. Channels where significant differences between conditions were found after FDR correction (*p* < 0.05) were indicated with red dots.

This analysis aimed to provide a detailed overview of the ERP wave behavior in congruent and incongruent conditions within each group separately. This approach allows for descriptive inferences about the functioning of each group, offering insight into their distinct neural processing patterns. Significant differences (*p* < 0.05) were found for the h-IPA at 300–500 and 1,050–1,200 ms, characterized by a higher positive amplitude for the congruent condition than for the incongruent condition, respectively. In both cases, these differences were widespread across anterior, posterior, and lateral electrodes. The results are displayed in Figures 2, 4, left panel). On the other hand, in the l-IPA group, significant differences between conditions were found only in the 500–700 ms interval over frontal scalp regions with higher negative amplitude for the congruent than incongruent condition. No other differences were observed in this group (see Figures 2, 4, right panel and Table 3).

## 4 Discussion

The present investigation aimed to study the association between the level of incidental physical activity and the ability to inhibit automatic responses in older adults, using event-related potentials to explore the neurobiological correlates of this relationship. Although our results confirm the presence of the Stroop effect in older adults, the expected behavioral differences, were not observed. However, the electrophysiological differences found between high and low IPA groups suggest variations in neural efficiency during the inhibition of automatic responses. These findings partially support our hypothesis, which proposed that incidental physical activity is associated with differences in the inhibition of automatic responses among cognitively healthy older adults.

Behavioral results confirmed the presence of a behavioral Stroop effect, marked by higher accuracy, faster reaction times, and lower reaction time variability in the congruent condition compared to the incongruent condition across both groups. This result aligns with previous research demonstrating that individuals, including older adults, exhibit slower reaction times and decreased accuracy in incongruent trials due to the increased cognitive demands required for conflict resolution (Aichert et al., 2012; Rey-Mermet and Gade, 2018; West and Alain, 1999, 2000).

Notably, at the behavioral level, no significant differences were observed between the h-IPA and l-IPA groups, apart from a marginally slower response time (*p* = 0.056) in the incongruent condition for the h-IPA group. While this difference was modest and not accompanied by improved accuracy, it may tentatively suggest that higher levels of IPA could be linked to a subtle tendency to favor accuracy over speed (Forstmann et al., 2011; Vallesi et al., 2021) when resolving cognitive conflict. However, this interpretation should be taken with caution, as the observed performance pattern does not clearly support a speed–accuracy trade-off and may also reflect random variation. Although we hypothesized that older adults with higher levels of incidental physical activity (IPA) would perform better in tasks requiring inhibition of automatic responses, this lack of difference may be explained by the fact that both groups underwent thorough evaluations to confirm their health status, ensuring that all participants met the required criteria. None of the participants showed evidence of cognitive disorders, and no significant differences were observed between the groups in cognitive, sociodemographic, blood analysis, or perceived quality of life and well-being measures. The sole difference between the groups was the level of IPA. Moreover, previous studies have shown that healthy participants with low levels of IPA (Alatorre-Cruz et al., 2020), and even healthy participants with electroencephalographic risk of cognitive disorder (Sánchez-Moguel et al., 2018), could perform as well at the behavioral level as those participants with high levels of IPA or without evidence of risk of cognitive disorder. The absence of differences between these groups is compatible with the presence of compensatory mechanisms that are evident only at the neural level (Cabeza, 2002; Reuter-Lorenz and Park, 2014; Stern, 2009), allowing subjects at greater risk to perform as well as subjects in the control groups.

Therefore, the evidence of the influence of IPA on older adults when an inhibition of the automatic response process is activated could become evident in the intermediate processes that take place between the detection of the stimulus and the emission of the motor response. Therefore, we propose that ERPs could be a more sensitive tool to explore them at the neural level. It is likely, then, that the results of the ERPs can provide insights that contribute to answering these questions and shed light on the relationship between IPA and inhibition in aging.

Regarding the ERP results, the first analysis compared the difference waves (incongruent minus congruent) between groups. When performing the counting-Stroop task, the groups only differed in their brain response at 1,050–1,200 ms over a large area on the scalp. This difference reflects the clear effect of the condition on this late negativity in the h-IPA group, in contrast to the l-IPA group which instead exhibited a positive deflection in the same time window. The negative amplitude observed in the 1,050–1,200 ms window for the incongruent respect to the congruent condition in the h-IPA group could be related to late-stage inhibitory processing post-execution likely related to re-evaluation and resolution of the conflict. Prior literature has linked late ERP negativities to response conflict monitoring (re-evaluation of the detection and resolution of competition between simultaneously active responses post-execution) and the evaluation of contextual memory information, particularly under high task demands (Herron, 2007; Johansson and Mecklinger, 2003; Swick et al., 2006). The absence of such a component in the l-IPA group, and, for instance, the presence of a positive deflection instead, may reflect a limited engagement of late-stage evaluative mechanisms. In contrast, the presence of this late negativity in the h-IPA group may indicate a more robust recruitment of these monitoring and re-evaluative processes to resolve residual interference from incongruent trials, even after responses are executed. This observation aligns with evidence suggesting that late ERP negativities reflect functionally heterogeneous processes, including action monitoring, uncertainty resolution, and sustained evaluation of retrieved episodes, which tend to be more prominently engaged under increased task difficulty. These findings align with the idea that rather than modulating early automatic filtering, IPA may be associated with enhanced capacity for sustained attentional control and re-evaluation of the conflict detection and resolution post-execution in the face of interference. However, this relationship may be more complex and require further exploration. While our initial framework proposed two distinct loci of inhibition, automatic and proactive mechanisms, our ERP results suggest that incidental physical activity may be more closely associated with enhanced reactive control processes, particularly those involved in late-stage processes post-execution.

Although no significant between-group differences were observed in ERP amplitudes within the 150–300, 300–500, and 500–700 ms time windows, exploratory within-group analyses revealed condition-specific neural activity in the h-IPA group that was not present in the l-IPA group. While not supporting direct between-group comparisons in early stages, these findings suggest differential engagement of inhibitory mechanisms. However, these patterns require cautious interpretation. Specifically, the h-IPA group appeared to employ early preparatory control to suppress automatic responses in anticipation of conflict, as suggested by the significant difference between conditions in the 300–500 ms window, which likely aligns with a proactive inhibitory mechanism (Xiao et al., 2011), an effect absent in the l-IPA group. Participants in the l-IPA group showed no evidence of early neural markers indicating conflict detection, suggesting a reduced or attenuated sensitivity to conflict at early processing stages. This diminished early conflict detection might result in less interference and less need to engage inhibitory control, which could explain the faster response times observed behaviorally in the l-IPA group in the incongruent condition (see Table 2). Importantly, although the behavioral data indicate that the l-IPA group detects conflicting information, the lack of early ERP condition effects suggests that this detection occurs later or is less intrusive neurally. This interpretation aligns with previous findings in individuals at electrophysiological risk for cognitive decline (Sánchez-Moguel et al., 2018). Later, the l-IPA group exhibited greater frontal positivity (500–700 ms) in the incongruent condition, suggesting delayed conflict resolution, consistent with West and Alain’s (1999) findings on late frontopolar positivity in classic Stroop tasks. This late positivity may reflect attentional resources (Ilan and Polich, 1999), greater allocation of resources for global decision-making and action planning (Koivisto and Revonsuo, 2003), or increased task difficulty (Kusak et al., 2000). These results could reflect the neurophysiological compensatory mechanisms enabling such cognitive processing to take place and allowing the l-IPA group to still accurately solve the interference task, as stated in the PASA model of brain aging (Davis et al., 2008). By the end of the analysis epoch (within the 1,050–1,200 ms window), the h-IPA group exhibited a significantly lower amplitude in the incongruent condition compared to the congruent condition, whereas the l-IPA group showed no difference between conditions. This result aligns with the findings from the initial between-groups analysis, where the difference waves between groups were compared and significant differences were found in this time window. In summary, these results suggest that the l-IPA group resolves conflict at a later stage, recruiting additional frontal resources, possibly as a compensatory mechanism, whereas the h-IPA group may resolve interference earlier.

The observed dissociation between behavioral (lack of significant differences between groups) and ERP markers further suggests that neural adaptations (e.g., enhanced conflict monitoring) may emerge before detectable behavioral changes. However, these preliminary interpretations require further investigation in future studies to confirm their validity.

Given that physical activity has been linked to enhanced executive function and neuroplasticity (Bherer et al., 2013; Erickson et al., 2015; Sofi et al., 2011), our findings support the notion that even unstructured daily movements may contribute to maintaining neurocognitive functioning in older adults.

Taking all these factors into account and considering the implications of this research for the field of cognitive reserve in aging, our findings provide novel insights into the relationship between IPA and cognitive reserve, emphasizing the importance of everyday physical activity in preserving executive function, particularly inhibitory control. Cognitive reserve has been widely recognized as a protective factor against age-related cognitive decline, enabling individuals to compensate for neuropathological changes and maintain functional independence (Barulli and Stern, 2013; Stern, 2009, 2012). While most studies have focused on structured exercise programs as a means of enhancing cognitive reserve (Colcombe and Kramer, 2003; Erickson et al., 2019; Sofi et al., 2011), our results suggest that engaging in more physical activity through every-day activities, such as walking, household tasks, and leisure may be related to better brain health. Given that IPA is more accessible and requires less effort than structured exercise, promoting IPA in daily routines may offer a practical and sustainable strategy for enhancing cognitive function in older adults, without diminishing the value of structured physical activity programs in improving overall health (Sanchez-Lopez et al., 2018). Among the limitations of this study are its cross-sectional design, which prevents the identification of causal conclusions, and the potential bias from self-reported IPA measures. Future research should incorporate objective IPA assessments and longitudinal designs to understand these effects better. Finally, although a non-probabilistic convenience sampling method was used, which may constrain the generalizability of our findings, the application of strict inclusion and exclusion criteria, along with the representation of participants across a range of incidental physical activity levels, supports the internal validity of the study.

### 4.1 Conclusion

This study highlights a potential association between incidental physical activity and the later post-execution stages of inhibitory control mechanisms at the neural level in older adults, as evaluated using a counting-Stroop task. In contrast to our hypothesis, while behavioral performance in the task did not reveal significant advantages for the high-IPA group compared to the low-IPA group, ERP analyses, which provide a more detailed insight into the processing of information, suggested different neural patterns associated with post-execution re-evaluation of response conflict detection and resolution in individuals with higher versus lower IPA levels, potentially linked to their engagement in incidental physical activity. These findings contribute to the growing body of literature on cognitive reserve and underscore the importance of promoting both structured and incidental physical activity as a means of supporting healthy neurocognitive aging.

## Data Availability

The raw data supporting the conclusions of this article will be made available by the authors, without undue reservation.

## References

[B1] Aichert D. S. Wöstmann N. M. Costa A. Macare C. Wenig J. R. Möller H.-J. (2012). Associations between trait impulsivity and prepotent response inhibition. *J. Clin. Exp. Neuropsychol.* 34 1016–1032. 10.1080/13803395.2012.706261 22888795

[B2] Alatorre-Cruz G. C. Sanchez-Lopez J. Silva-Pereyra J. Fernández T. (2020). Effects of incidental physical activity on morphosyntactic processing in aging. *PLoS One* 15:0239727. 10.1371/journal.pone.0239727 32991617 PMC7523944

[B3] AMAI. (2011). *Cuestionario Regla AMAI NSE 8 × 7.* Asociación Mexicana de Agencias de Investigación de Mercados y Opinión Pública, A.C. Mexico City.

[B4] Andrews S. C. Parekh D. Brady B. Delbaere K. Hamidul Huque M. Killcross S. (2022). Associations between planned exercise, walking, incidental physical activity, and habit strength in older people: A cross-sectional study. *J. Aging Phys. Activity* 30 813–823. 10.1123/japa.2021-0284 34929661

[B5] Aron A. R. (2011). From reactive to proactive and selective control: developing a richer model for stopping inappropriate responses. *Biol. Psychiatry* 69 e55–e68. 10.1016/j.biopsych.2010.07.024 20932513 PMC3039712

[B6] Aron A. R. Robbins T. W. Poldrack R. A. (2004). Inhibition and the right inferior frontal cortex. *Trends Cogn. Sci.* 8 170–177. 10.1016/j.tics.2004.02.010 15050513

[B7] Bari A. Robbins T. W. (2013). Inhibition and impulsivity: Behavioral and neural basis of response control. *Prog. Neurobiol.* 108 44–79. 10.1016/j.pneurobio.2013.06.005 23856628

[B8] Barnes J. N. Pearson A. G. Corkery A. T. Eisenmann N. A. Miller K. B. (2021). Exercise, arterial stiffness, and cerebral vascular function: Potential impact on brain health. *J. Int. Neuropsychol. Soc.* 27 761–775. 10.1017/S1355617721000394 33952365 PMC8496967

[B9] Barulli D. Stern Y. (2013). Efficiency, capacity, compensation, maintenance, plasticity: Emerging concepts in cognitive reserve. *Trends Cogn. Sci.* 17 502–509. 10.1016/j.tics.2013.08.012 24018144 PMC3840716

[B10] Bherer L. Erickson K. I. Liu-Ambrose T. (2013). A review of the effects of physical activity and exercise on cognitive and brain functions in older adults. *J. Aging Res.* 2013 657508. 10.1155/2013/657508 24102028 PMC3786463

[B11] Blinkouskaya Y. Caçoilo A. Gollamudi T. Jalalian S. Weickenmeier J. (2021). Brain aging mechanisms with mechanical manifestations. *Mech. Ageing Development* 200:111575. 10.1016/j.mad.2021.111575 34600936 PMC8627478

[B12] Braver T. S. Paxton J. L. Locke H. S. Barch D. M. (2009). Flexible neural mechanisms of cognitive control within human prefrontal cortex. *Proc. Natl. Acad. Sci.* 106 7351–7356. 10.1073/pnas.0808187106 19380750 PMC2678630

[B13] Bush G. Whalen P. J. Rosen B. R. Jenike M. A. McInerney S. C. Rauch S. L. (1998). The counting stroop: An interference task specialized for functional neuroimaging-validation study with functional MRI. *Hum. Brain Mapp.* 6 270–282. 10.1002/(SICI)1097-019319986:4<270::AID-HBM6<3.0.CO;2-0 9704265 PMC6873370

[B14] Cabeza R. (2002). Hemispheric asymmetry reduction in older adults: The HAROLD model. *Psychol. Aging* 17 85–100. 10.1037/0882-7974.17.1.85 11931290

[B15] Cabeza R. Albert M. Belleville S. Craik F. I. M. Duarte A. Grady C. L. (2018). Maintenance, reserve and compensation: The cognitive neuroscience of healthy ageing. *Nat. Rev. Neurosci.* 19 701–710. 10.1038/s41583-018-0068-2 30305711 PMC6472256

[B16] Carlson M. C. Hasher L. Connelly S. L. Zacks R. T. (1995). Aging, Distraction, and the benefits of predictable location. *Psychol. Aging* 10 427–436. 10.1037/0882-7974.10.3.427 8527063

[B17] Chen C. Nakagawa S. (2023). Physical activity for cognitive health promotion: An overview of the underlying neurobiological mechanisms. *Ageing Res. Rev.* 86:101868. 10.1016/j.arr.2023.101868 36736379

[B18] Colcombe S. Kramer A. F. (2003). Fitness effects on the cognitive function of older adults: A meta-analytic study. *Psychol. Sci.* 14 125–130. 10.1111/1467-9280.t01-1-01430 12661673

[B19] Davis S. W. Dennis N. A. Daselaar S. M. Fleck M. S. Cabeza R. (2008). Que PASA? The posterior-anterior shift in aging. *Cereb. Cortex* 18 1201–1209. 10.1093/cercor/bhm155 17925295 PMC2760260

[B20] De Abajo S. Larriba R. Marquez S. (2001). Validity and reliability of the yale physical activity survey in spanish elderly. *J. Sports Med. Phys. Fitness* 41 479–485. 11687767

[B21] Delorme A. Makeig S. (2004). EEGLAB: An open source toolbox for analysis of single-trial EEG dynamics including independent component analysis. *J. Neurosci. Methods* 134 9–21. 10.1016/j.jneumeth.2003.10.009 15102499

[B22] Diamond A. (2013). Executive functions. *Annu. Rev. Psychol*. 64 135–168. 10.1146/annurev-psych-113011-143750 23020641 PMC4084861

[B23] Endicott J. Nee J. Harrison W. Blumenthal R. (1993). Quality of life enjoyment and satisfaction questionnaire: A new measure. *Psychopharmacol. Bull.* 29 321–326. 8290681

[B24] Enokida T. Hattori K. Maeda C. Tomizawa T. Kunugi H. (2024). Association between overweight and central interleukin-6 in a nonclinical adult population. *Neuropsychopharmacol. Rep.* 44 835–841. 10.1002/npr2.12488 39401137 PMC11609746

[B25] Erickson K. I. Hillman C. H. Kramer A. F. (2015). Physical activity, brain, and cognition. *Curr. Opin. Behav. Sci.* 4 27–32. 10.1016/j.cobeha.2015.01.005

[B26] Erickson K. I. Hillman C. Stillman C. M. Ballard R. M. Bloodgood B. Conroy D. E. (2019). Physical activity, cognition, and brain outcomes: A review of the 2018 physical activity guidelines. *Med. Sci. Sports Exerc.* 51 1242–1251. 10.1249/MSS.0000000000001936 31095081 PMC6527141

[B27] Erickson K. I. Voss M. W. Prakash R. S. Basak C. Szabo A. Chaddock L. (2011). Exercise training increases size of hippocampus and improves memory. *Proc. Natl. Acad. Sci.* 108 3017–3022. 10.1073/pnas.1015950108 21282661 PMC3041121

[B28] Feng J. Song H. Wang Y. Zhou Q. Zhou C. Jin J. (2024). Exploring the relationship between physical activity and cognitive function: An fMRI pilot study in young and older adults. *Front. Public Health* 12:1413492. 10.3389/fpubh.2024.1413492 39091524 PMC11291347

[B29] Forstmann B. U. Tittgemeyer M. Wagenmakers E.-J. Derrfuss J. Imperati D. Brown S. (2011). The speed-accuracy tradeoff in the elderly brain: A structural model-based approach. *J. Neurosci.* 31 17242–17249. 10.1523/JNEUROSCI.0309-11.2011 22114290 PMC6623864

[B30] Franceschi C. Campisi J. (2014). Chronic inflammation (inflammaging) and its potential contribution to age-associated diseases. *J. Gerontol. Ser. Biol. Sci. Med. Sci.* 69 S4–S9. 10.1093/gerona/glu057 24833586

[B31] Frank P. Kaushal A. Poole L. Lawes S. Chalder T. Cadar D. (2019). Systemic low-grade inflammation and subsequent depressive symptoms: Is there a mediating role of physical activity? *Brain Behav. Immun.* 80 688–696. 10.1016/j.bbi.2019.05.017 31085217

[B32] Gajewski P. D. Falkenstein M. (2015). Long-term habitual physical activity is associated with lower distractibility in a Stroop interference task in aging: Behavioral and ERP evidence. *Brain Cogn.* 98 87–101. 10.1016/j.bandc.2015.06.004 26160263

[B33] Gajewski P. D. Falkenstein M. Thönes S. Wascher E. (2020). Stroop task performance across the lifespan: High cognitive reserve in older age is associated with enhanced proactive and reactive interference control. *NeuroImage* 207:116430. 10.1016/j.neuroimage.2019.116430 31805383

[B34] Gongora-Meza L. F. Sanchez-Lopez J. (2025). Incidental physical activity and physical fitness associate with sustained attention and impulse control in older adults. *Ageing Int.* 50:2. 10.1007/s12126-024-09580-x

[B35] Gratton G. Coles M. G. H. Donchin E. (1983). A new method for off-line removal of ocular artifact. *Electroencephalogr. Clin Neurophysiol.* 55 468–484. 10.1016/0013-4694(83)90135-9 6187540

[B36] Groppe D. M. Urbach T. P. Kutas M. (2011). Mass univariate analysis of event-related brain potentials/fields I: A critical tutorial review. *Psychophysiology* 48 1711–1725. 10.1111/j.1469-8986.2011.01273.x 21895683 PMC4060794

[B37] Hasher L. Stoltzfus E. R. Zacks R. T. Rypma B. (1991). Age and inhibition. *J. Exp. Psychol. Learn. Mem. Cogn.* 17 163–169. 10.1037/0278-7393.17.1.163 1826730

[B38] Herron J. E. (2007). Decomposition of the ERP late posterior negativity: Effects of retrieval and response fluency. *Psychophysiology* 44 233–244. 10.1111/j.1469-8986.2006.00489.x 17343707

[B39] Ilan A. B. Polich J. (1999). P300 and response time from a manual Stroop task. *Clin. Neurophysiol.* 110 367–373. 10.1016/S0168-5597(98)00053-7 10210626

[B40] Ingold M. Tulliani N. Chan C. C. H. Liu K. P. Y. (2020). Cognitive function of older adults engaging in physical activity. *BMC Geriat.* 20:229. 10.1186/s12877-020-01620-w 32616014 PMC7333382

[B41] Itti L. Koch C. (2001). Computational modelling of visual attention. *Nat. Rev. Neurosci.* 2 194–203. 10.1038/35058500 11256080

[B42] Jintian L. (2019). Influence of physical activity on executive control function in older adults by event-related potentials. *Chinese J. Tissue Eng. Res.* 23:1693. 10.3969/j.issn.2095-4344.1137

[B43] Johansson M. Mecklinger A. (2003). The late posterior negativity in ERP studies of episodic memory: Action monitoring and retrieval of attribute conjunctions. *Biol. Psychol.* 64 91–117. 10.1016/S0301-0511(03)00104-2 14602357

[B44] Kipp-Harnishfeger K. (1995). “The development of cognitive inhibition,” in *In Interference and Inhibition in Cognition*, eds Dempster F. N. Brainerd C. J. (Amsterdam: Elsevier), 175–204. 10.1016/B978-012208930-5/50007-6

[B45] Koivisto M. Revonsuo A. (2003). An ERP study of change detection, change blindness, and visual awareness. *Psychophysiology* 40 423–429. 10.1111/1469-8986.00044 12946115

[B46] Kusak G. Grune K. Hagendorf H. Metz A.-M. (2000). Updating of working memory in a running memory task: An event-related potential study. *Int. J. Psychophysiol.* 39 51–65. 10.1016/S0167-8760(00)00116-1 11120347

[B47] Larson M. J. Clayson P. E. Clawson A. (2014). Making sense of all the conflict: A theoretical review and critique of conflict-related ERPs. *Int. J. Psychophysiol.* 93 283–297. 10.1016/j.ijpsycho.2014.06.007 24950132

[B48] Littman R. Takács Á (2017). Do all inhibitions act alike? A study of go/no-go and stop-signal paradigms. *PLoS One* 12:e0186774. 10.1371/journal.pone.0186774 29065184 PMC5655479

[B49] Logan G. D. (1985). On the ability to inhibit simple thoughts and actions: II. Stop-signal studies of repetition priming. *J. Exp. Psychol. Learn. Mem. Cogn.* 11 675–691. 10.1037/0278-7393.11.1-4.675

[B50] López Sánchez J. Granados D. (2021). Cognitive reserve correlates with task-related EEG power in healthy aging. *IOSR J. Nurs. Health Sci.* 10 39–47. 10.9790/1959-1004073947

[B51] Luck S. J. Gaspelin N. (2017). How to get statistically significant effects in any ERP experiment (and why you shouldn’t). *Psychophysiology* 54 146–157. 10.1111/psyp.12639 28000253 PMC5178877

[B52] Martini M. Enoch J. Kramer A. F. (2024). The effects of a short exercise bout on executive functions in healthy older adults. *Sci. Rep.* 14 28827. 10.1038/s41598-024-79685-5 39572743 PMC11582581

[B53] McDonald J. H. (2009). *Handbook of Biological Statistics.* Baltimore, MA: Sparky House Publishing.

[B54] Meng X. D’Arcy C. (2012). Education and dementia in the context of the cognitive reserve hypothesis: A systematic review with meta-analyses and qualitative analyses. *PLoS One* 7:e38268. 10.1371/journal.pone.0038268 22675535 PMC3366926

[B55] Nishida Y. Tanaka K. Hara M. Hirao N. Tanaka H. Tobina T. (2015). Effects of home-based bench step exercise on inflammatory cytokines and lipid profiles in elderly Japanese females: A randomized controlled trial. *Arch. Gerontol. Geriatr.* 61 443–451. 10.1016/j.archger.2015.06.017 26228714

[B56] Nithianantharajah J. Hannan A. J. (2009). The neurobiology of brain and cognitive reserve: Mental and physical activity as modulators of brain disorders. *Prog. Neurobiol.* 89 369–382. 10.1016/j.pneurobio.2009.10.001 19819293

[B57] Ostrosky-Solis F. Ardila A. Rosselli M. (1999). NEUROPSI: a brief neuropsychological test battery in Spanish with norms by age and educational level. *J. Int. Neuropsychol. Soc.* 5 413–433. 10.1017/S1355617799555045 10439587

[B58] Pernet C. R. Latinus M. Nichols T. E. Rousselet G. A. (2015). Cluster-based computational methods for mass univariate analyses of event-related brain potentials/fields: A simulation study. *J. Neurosci. Methods* 250 85–93. 10.1016/j.jneumeth.2014.08.003 25128255 PMC4510917

[B59] Portugal A. C. A. Afonso A. S. Caldas A. L. Maturana W. Mocaiber I. Machado-Pinheiro W. (2018). Inhibitory mechanisms involved in Stroop-matching and stop-signal tasks and the role of impulsivity. *Acta Psychol.* 191 234–243. 10.1016/j.actpsy.2018.10.003 30343096

[B60] Raichlen D. A. Alexander G. E. (2017). Adaptive capacity: An evolutionary neuroscience model linking exercise, cognition, and brain health. *Trends Neurosci.* 40 408–421. 10.1016/j.tins.2017.05.001 28610948 PMC5926798

[B61] Reuter-Lorenz P. A. Park D. C. (2014). How does it STAC up? Revisiting the scaffolding theory of aging and cognition. *Neuropsychol. Rev*. 24 355–370. 10.1007/s11065-014-9270-9 25143069 PMC4150993

[B62] Rey-Mermet A. Gade M. (2018). Inhibition in aging: What is preserved? What declines? A meta-analysis. *Psychon. Bull. Rev.* 25 1695–1716. 10.3758/s13423-017-1384-7 29019064

[B63] Rey-Mermet A. Gade M. Oberauer K. (2018). Should we stop thinking about inhibition? Searching for individual and age differences in inhibition ability. *J. Exp. Psychol. Learn. Mem. Cogn.* 44 501–526. 10.1037/xlm0000450 28956944

[B64] Ross R. McGuire K. A. (2011). Incidental physical activity is positively associated with cardiorespiratory fitness. *Med. Sci. Sports Exerc.* 43 2189–2194. 10.1249/MSS.0b013e31821e4ff2 21502894

[B65] Sanchez-Lopez J. Silva-Pereyra J. Fernández T. Alatorre-Cruz G. C. Castro-Chavira S. A. González-López M. (2018). High levels of incidental physical activity are positively associated with cognition and EEG activity in aging. *PLoS One* 13:e0191561. 10.1371/journal.pone.0191561 29370215 PMC5784952

[B66] Sánchez-Moguel S. M. Alatorre-Cruz G. C. Silva-Pereyra J. González-Salinas S. Sanchez-Lopez J. Otero-Ojeda G. A. (2018). Two different populations within the healthy elderly: Lack of conict detection in those at risk of cognitive decline. *Front. Hum. Neurosci.* 11:658. 10.3389/fnhum.2017.00658 29375352 PMC5768990

[B67] Shephard R. J. (2013). A critique of RPE as a basis of exercise prescription. *Eur. J. Appl. Physiol.* 113 1369–1370. 10.1007/s00421-013-2630-y 23539440

[B68] Simpson A. Carroll D. J. (2019). Understanding early inhibitory development: Distinguishing two ways that children use inhibitory control. *Child Development* 90 1459–1473. 10.1111/cdev.13283 31286502

[B69] Sofi F. Valecchi D. Bacci D. Abbate R. Gensini G. F. Casini A. (2011). Physical activity and risk of cognitive decline: A meta-analysis of prospective studies: Physical activity and risk of cognitive decline. *J. Internal Med.* 269 107–117. 10.1111/j.1365-2796.2010.02281.x 20831630

[B70] Stern Y. (2002). What is cognitive reserve? Theory and research application of the reserve concept. *J. Int. Neuropsychol. Soc.* 8 448–460. 10.1017/S1355617702813248 11939702

[B71] Stern Y. (2009). Cognitive reserve?. *Neuropsychologia* 47 2015–2028. 10.1016/j.neuropsychologia.2009.03.004 19467352 PMC2739591

[B72] Stern Y. (2012). Cognitive reserve in ageing and Alzheimer’s disease. *Lancet Neurol.* 11 1006–1012. 10.1016/S1474-4422(12)70191-6 23079557 PMC3507991

[B73] Stern Y. (2017). An approach to studying the neural correlates of reserve. *Brain Imaging Behav.* 11 410–416. 10.1007/s11682-016-9566-x 27450378 PMC5810375

[B74] Stern Y. Arenaza-Urquijo E. M. Bartrés-Faz D. Belleville S. Cantilon M. Chetelat G. (2020). Whitepaper: Defining and investigating cognitive reserve, brain reserve, and brain maintenance. *Alzheimer’s Dement.* 16 1305–1311. 10.1016/j.jalz.2018.07.219 30222945 PMC6417987

[B75] Swick D. Senkfor A. J. Van Petten C. (2006). Source memory retrieval is affected by aging and prefrontal lesions: Behavioral and ERP evidence. *Brain Res.* 1107 161–176. 10.1016/j.brainres.2006.06.013 16828722 PMC2365725

[B76] Tucker A. M. Stern Y. (2011). Cognitive reserve in aging. *Curr. Alzheimer Res.* 8 354–360. 10.2174/156720511795745320 21222591 PMC3135666

[B77] Valenzuela M. J. Sachdev P. (2006). Brain reserve and cognitive decline: A non-parametric systematic review. *Psychol. Med.* 36 1065–1073. 10.1017/S0033291706007744 16650343

[B78] Vallesi A. Tronelli V. Lomi F. Pezzetta R. (2021). Age differences in sustained attention tasks: A meta-analysis. *Psychon. Bull. Rev.* 28 1755–1775. 10.3758/s13423-021-01908-x 33772477 PMC8642381

[B79] Vinetti G. Mozzini C. Desenzani P. Boni E. Bulla L. Lorenzetti I. (2015). Supervised exercise training reduces oxidative stress and cardiometabolic risk in adults with type 2 diabetes: A randomized controlled trial. *Sci. Rep.* 5:9238. 10.1038/srep09238 25783765 PMC4363871

[B80] Wärnberg J. Cunningham K. Romeo J. Marcos A. (2010). Physical activity, exercise and low-grade systemic inflammation. *Proc. Nutr. Soc.* 69 400–406. 10.1017/S0029665110001928 20598198

[B81] Wechsler D. (2012). *Wechsler Adult Intelligence Scale–Fourth Edition (WAIS-IV), Administration Manual.* Mexico City: Manual Moderno.

[B82] West R. Alain C. (1999). Event-related neural activity associated with the Stroop task. *Cogn. Brain Res.* 8 157–164. 10.1016/S0926-6410(99)00017-8 10407204

[B83] West R. Alain C. (2000). Age-related decline in inhibitory control contributes to the increased Stroop effect observed in older adults. *Psychophysiology* 37 179–189. 10.1111/1469-8986.3720179 10731768

[B84] Xiao X. Yang W. Jia L. Lei M. Chen A. Zhang Q. (2011). Neural mechanism of conflict control in a number interference task. *NeuroReport* 22 979–983. 10.1097/WNR.0b013e32834d8853 22045253

[B85] Zhang R. Geng X. Lee T. M. C. (2017). Large-scale functional neural network correlates of response inhibition: An fMRI meta-analysis. *Brain Struct. Funct.* 222 3973–3990. 10.1007/s00429-017-1443-x 28551777 PMC5686258

[B86] Zhang W. Zhou C. Chen A. (2023). A systematic review and meta-analysis of the effects of physical exercise on white matter integrity and cognitive function in older adults. *GeroScience* 46 2641–2651. 10.1007/s11357-023-01033-8 38108993 PMC10828294

